# Thymic stromal lymphopoietin improves protective immunity of the SARS-CoV-2 subunit vaccine by inducing dendritic cell-dependent germinal center response

**DOI:** 10.1128/jvi.02323-24

**Published:** 2025-03-04

**Authors:** Hao Hu, Ying Zhang, Housheng Zheng, Xiwen Zhao, Wei Ran, Chenghui Liao, Mingcheng Lu, Jian Zhou, Xun Song, Liang Ye

**Affiliations:** 1 Department of Immunology, International Cancer Center, Shenzhen University Medical School, Shenzhen University 718776 , Shenzhen, China; 2 Guangdong Key Laboratory for Biomedical Measurements and Ultrasound Imaging, National Regional Key Technology Engineering Laboratory for Medical Ultrasound, School of Biomedical Engineering, Shenzhen University 620599 , Shenzhen, China; 3 The First Affiliated Hospital of Guangzhou Medical University Guangzhou Medical University 26468 , Guangzhou, China; 4 College of Pharmacy Shenzhen Technology University 507738 , Shenzhen, China; Loyola University Chicago - Health Sciences Campus, Maywood, Illinois, USA

**Keywords:** vaccination, thymic stromal lymphopoietin, dendritic cells, germinal center response, neutralizing antibody, SARS-CoV-2

## Abstract

To effectively control viral pandemics, a multifaceted approach incorporating diverse vaccination strategies may be imperative. Our study demonstrates that both mucosal and non-mucosal immunization of mice with severe acute respiratory syndrome coronavirus 2 (SARS-CoV-2) subunit vaccine enriched with thymic stromal lymphopoietin (TSLP) results in potent vaccine-specific systemic IgG and IgG1 antibody responses, but only mucosal immunization can induce robust mucosal IgA antibody and neutralizing antibody against SARS-CoV-2 wild-type, Alpha B.1.1.7, and Delta B.1.617.2 variant strains. We found that the immune-enhancing effect of TSLP relies on promoting dendritic cell (DC) migration and activation, which in turn enhances the germinal center (GC) responses. Furthermore, intranasal administration of the TSLP-adjuvanted vaccine protected against challenges from both SARS-CoV-2 wild-type and Delta B.1.617.2 strains. Our findings offer valuable insights into the importance of TSLP as a critical mucosal adjuvant in enhancing mucosal adaptive immunity by modulating DC-GC crosstalk, hence enhancing vaccination effectiveness.

**IMPORTANCE:**

The current development and implementation of subunit vaccines in clinical practice encounter a number of challenges. The most significant difficulties are the scarcity of effective adjuvants and the fact that most vaccination programs rely on non-mucosal injections, which frequently fail to establish mucosal adaptive immunity and hence restrict the potential to protect against respiratory viral infections. Our research indicates that TSLP can effectively boost SARS-CoV-2 subunit vaccine-specific systemic and mucosal humoral immunity in mice after intranasal immunization, protecting them from SARS-CoV-2 wild-type and Delta variant infection. The mucosal adjuvant effects of TSLP depend on DC migration and activation, which boosts germinal center responses. Therefore, supplementation of mucosal subunit vaccines with TSLP should be considered in vaccine development, particularly when the vaccine is administered to children and the elderly. Our findings provide new evidence for the development of mucosal adjuvants for infectious diseases, potentially facilitating future vaccine development.

## INTRODUCTION

Vaccination is one of the most cost-effective and efficient strategies to prevent viral infection (1). However, the immune responses induced by subunit vaccines are frequently insufficient, particularly in young and elderly individuals with weakened immune systems, which can significantly compromise the effectiveness of the vaccine (2). Adjuvants, as a key component of a subunit vaccine, can significantly improve the immunogenicity of the vaccine and protect against viral infections (3, 4). However, clinically approved adjuvants are very limited, which increases immune response through intramuscular or subcutaneous immunization but cannot effectively activate mucosal adaptive immunity (4–7). Therefore, the development of adjuvants capable of eliciting potent systemic and mucosal adaptive immune responses is anticipated to enhance the efficacy of viral vaccines.

Thymic stromal lymphopoietin (TSLP) is an interleukin-7 (IL-7) cytokine-family cytokine mainly produced by epithelial cells and dendritic cells (DCs) (8, 9). TSLP plays a pivotal role in modulating immune responses by engaging with its specific receptor complex, a heterodimer composed of the TSLP receptor (TSLPR) and the IL-7 receptor alpha chain (IL-7Rα), thereby initiating downstream signaling pathways (9, 10). TSLP stimulates a wide range of immune cells, including dendritic cells (DCs), T cells, and B cells (8, 11–14). Human TSLP can induce T follicular helper (Tfh) cell differentiation and IgE production by stimulating DC cells expressing OX40 ligand (15). TSLP is regarded as a vital immunoregulatory factor, with its abnormal expression or dysfunction potentially linked to the onset and progression of various immune-related diseases (8, 9, 16, 17).

We and other research groups reported that mouse TSLP acts as a novel adjuvant (18–20). Intranasal administration of TSLP with the human immunodeficiency virus (HIV) vaccine gp140 elicits robust and sustained humoral and cellular immune responses (18). Our previous studies have shown that mice intranasally or rectally immunized with TSLP and universal influenza vaccines can boost serum vaccine-specific systemic IgG1 and mucosal IgA levels and effectively resist influenza virus challenge in a TSLPR-dependent manner (19, 21, 22). TSLP signaling is also required for virus-specific CD8^+^ T cell responses when a live-attenuated influenza vaccine is administered intranasally (19, 20). Although the adjuvant role of TSLP has been identified, the optimal administration route and mechanism of action to exert its adjuvant activity remain unknown.

In this study, we investigated the role of TSLP in the adaptive immune response after immunization with the severe acute respiratory syndrome coronavirus 2 (SARS-CoV-2) subunit vaccine by three distinct immunization routes. We found that intranasal, intramuscular, and intraperitoneal administration of SARS-CoV-2 subunit vaccines supplemented with TSLP can induce strong vaccine-specific IgG and IgG1 antibodies, whereas only intranasal immunization induces strong mucosal IgA antibodies and neutralizing antibodies against SARS-CoV-2 wild-type and mutant strains, implying that mucosal immunization is particularly important for exerting the adjuvant effect of TSLP. Mechanistically, TSLP enhances mucosal adaptive immunity primarily by acting on dendritic cells (cDCs) to induce their activation and migration, which promotes T follicular helper (Tfh) cell and germinal center (GC) B cell responses. Intranasal immunization with the SARS-CoV-2 subunit vaccine supplemented with TSLP can effectively protect mice against infection by SARS-CoV-2 wild-type and Delta B.1.617.2 strains. Therefore, our data revealed that TSLP enhances mucosal adaptive immunity, which is critical for improving vaccine efficacy and resisting SARS-CoV-2 infection.

## RESULTS

### TSLP boosts SARS-CoV-2 vaccine-specific IgG1 and IgA antibody production upon intranasal vaccination

**Fig 1:**
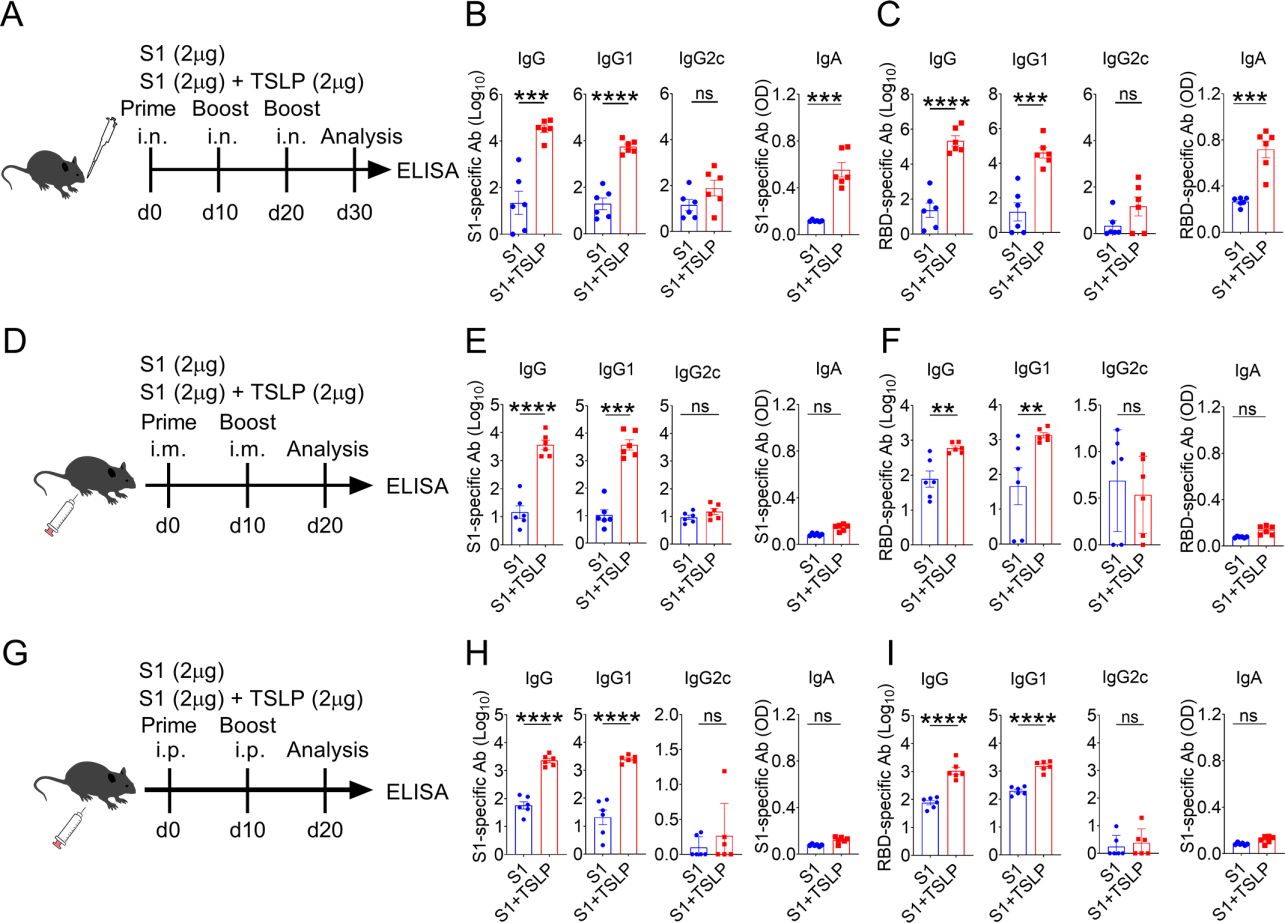
TSLP promotes antibody production when the SARS-CoV-2 vaccine is delivered by different immunization routes. (**A**) WT mice were immunized by the intranasal (i.n.) route with 2 µg SARS-CoV-2 S1 vaccine in the presence or absence of 2 µg TSLP. Booster immunizations were performed 10 days and 20 days later. (**B-C**) Ten days after the second booster immunization, titers of serum S1-specific and RBD-specific IgG subclasses and BALF IgA levels were determined by ELISA. (**D**) WT mice were immunized by the intramuscular (i.m.) route with 2 µg SARS-CoV-2 S1 vaccine in the presence or absence of 2 µg TSLP; a booster immunization was performed 10 days later. (**E-F**) Ten days after the booster immunization, titers of serum S1-specific and RBD-specific IgG subclasses and BALF IgA levels were determined by ELISA. (**G**) WT mice were immunized by the intraperitoneal (i.p.) route with 2 µg SARS-CoV-2 S1 vaccine in the presence or absence of 2 µg TSLP; a booster immunization was performed 10 days later. (**H-I**) Ten days after the booster immunization, titers of serum S1-specific and RBD-specific IgG subclasses and BALF IgA levels were determined by ELISA. Each symbol represents an individual animal, *n* = 6 mice per group. Data are shown as mean ± SEM. **P < 0.01, ***P < 0.001, ****P < 0.0001, ns, no significant difference, by unpaired two-tailed Student’s *t*-test.

Thymic stromal lymphopoietin (TSLP) has been identified as a novel adjuvant (18–20), but the most efficient vaccination route for exerting its adjuvant effect has yet to be determined. To address this issue, we immunized C57BL/6 mice (designated wild-type mice, WT mice) using three distinct immunization routes, administering either 2 µg of SARS-CoV-2 spike protein S1 subunit (S1) alone or in combination with 2 µg of TSLP. As shown in Fig. 1A or SARS-CoV-2 receptor-binding domain (RBD)-specific IgG subclasses in serum and S1- or RBD-specific IgA in bronchoalveolar lavage fluid (BALF) were measured by enzyme-linked immunosorbent assay (ELISA) 10 days after the second booster intranasal immunization. We found that intranasal administration of the S1 vaccine alone resulted in poor S1- or RBD-specific serum IgG and IgG1 and BALF IgA production, whereas S1 vaccine supplementation with TSLP enhanced S1- or RBD-specific serum IgG and IgG1 and BALF IgA levels (Fig. 1B and C). However, TSLP failed to boost S1- or RBD-specific serum IgG2c production (Fig. 1B and C). These results suggested that TSLP is important for inducing vaccine-specific systemic IgG1 and mucosal IgA production after intranasal immunization.

To determine if TSLP acts as an adjuvant through non-mucosal route immunization, WT mice received S1 with or without TSLP via intramuscular (Fig. 1D) or intraperitoneal (Fig. 1G) injection, and serum and BALF S1 or RBD-specific antibody production was evaluated by ELISA after boost immunization. With intramuscular vaccination, TSLP boosted the levels of serum IgG and IgG1, but not IgG2c specific to S1 or RBD (Fig. 1E and F), whereas the levels of BALF IgA remained unchanged (Fig. 1E and F). Consistent with these findings, TSLP improved S1- or RBD-specific serum IgG and IgG1 without altering BALF IgA production through intraperitoneal immunization (Fig. 1H and I). These data demonstrate that mucosal immunization is the most effective approach for TSLP to exert its adjuvant effect, as it induces both systemic and mucosal humoral immune responses, which are critical for protection against respiratory tract infections.

### TSLP induces neutralizing antibodies against SARS-CoV-2 and its variant strains by intranasal immunization

**Fig 2:**
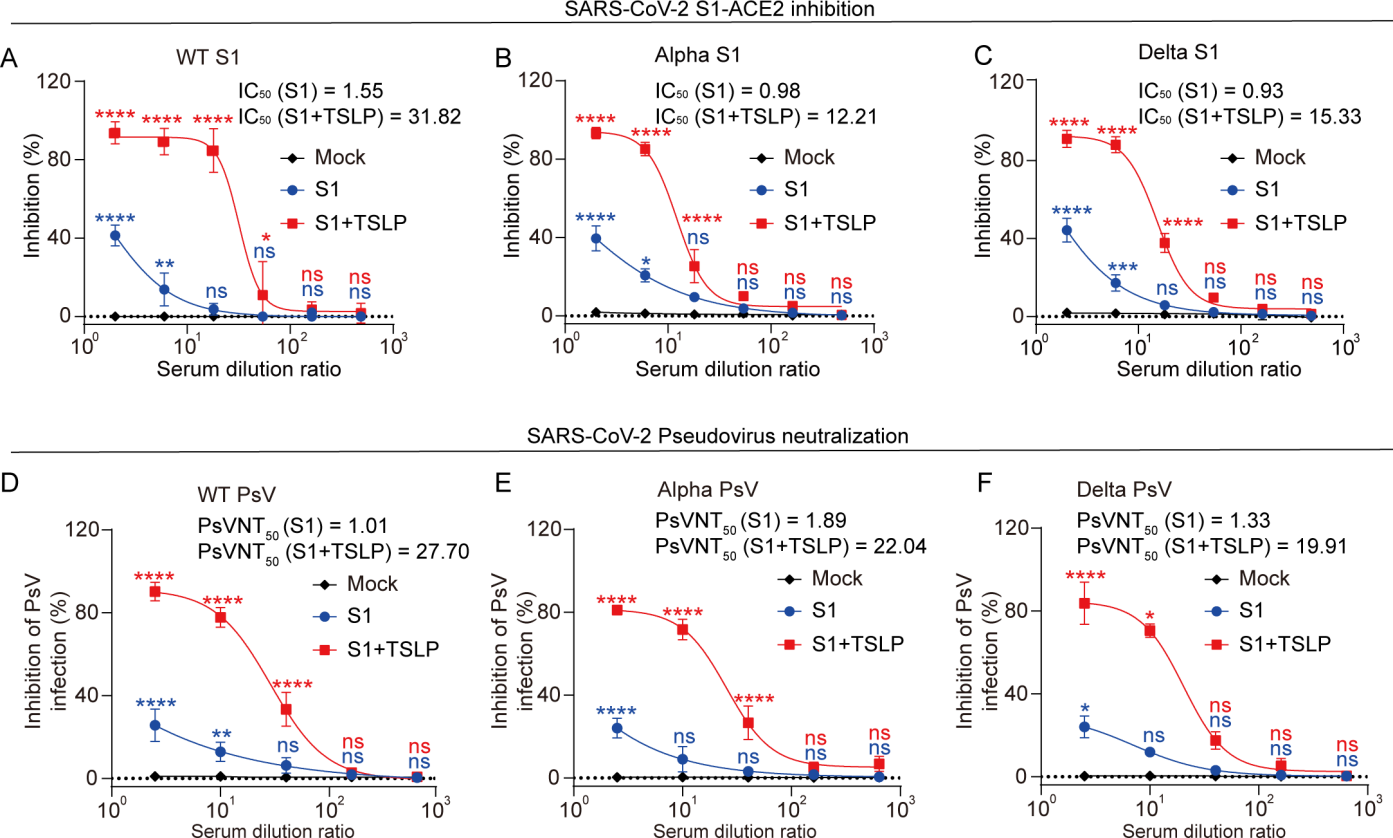
TSLP enhances SARS-CoV-2 vaccine-induced neutralizing antibody production after intranasal immunization. WT mice were immunized with SARS-CoV-2 S1 (2 µg) in the presence or absence of TSLP (2 µg) via the intranasal route. Booster immunizations were performed 10 days and 20 days later. Serum was collected 10 days after the second booster immunization. (**A-C**) Competitive ELISA was used to determine the percent inhibition of SARS-CoV-2 (WT) S1 (**A**), Alpha B.1.1.7 S1 (**B**), or Delta B.617.2 S1 (**C**) binding to hACE2 in the presence of serially diluted serum (1:2, 1:6, 1:18, 1:54, 1:162, and 1:486 dilution) from unimmunized mice (Mock, black color) or immunized mice with S1 (blue color) or S1 + TSLP (red color). (**D-E**) The percent inhibition of serially diluted serum (1:2.5, 1:10, 1:40, 1:160, and 1:640 dilution) from unimmunized mice (Mock, black color) or immunized mice with S1 (blue color) or S1 + TSLP (red color) to the SARS-CoV-2 WT (**D**), Alpha B.1.1.7 (**E**), or Delta B.617.2 (**F**) pseudoviruses was assessed by the SARS-CoV-2 pseudovirus neutralization assay. Data are shown as mean ± SD. *P < 0.05, **P < 0.01, ***P < 0.001, ****P < 0.0001, ns, no significant difference, by two-way ANOVA with Dunnett’s multiple-comparison test. Red asterisks indicate statistically significant differences between S1 and S1 + TSLP groups; blue asterisks indicate differences between S1 and mock groups. The 50% inhibitory concentration (IC_50_) (**A-C**) and 50% pseudovirus neutralization titer (PsVNT_50_) (**D-F**) were calculated by sigmoidal curve fitting with four-parameter nonlinear regression using GraphPad Prism v.8.0 software.

Neutralizing antibodies are critical for controlling SARS-CoV-2 infection (23–26). To determine whether TSLP can induce robust neutralizing antibody responses after immunization, we employed a competitive ELISA to evaluate serum from S1 and S1 combined with TSLP-immunized mice against the S1 and human receptor angiotensin-converting enzyme 2 (hACE2) interactions. Our results demonstrated that serum from mice intranasally immunized with S1 enriched with TSLP effectively inhibits SARS-CoV-2 wild-type (WT) S1 and hACE2 binding with half-maximal inhibitory concentration (IC_50_) values of 31.82, whereas serum from mice immunized with S1 alone exhibits weak blocking effect on SARS-CoV-2 WT S1 binding to hACE2 with IC_50_ values of 1.55 (Fig. 2A). However, the enhancing effect of TSLP on neutralizing antibodies was not observed in mice immunized intraperitoneally and intramuscularly (Fig. S1A and B). More importantly, the immune serum from intranasal immunization of the S1 vaccine containing TSLP exhibited strong inhibition of the interaction between SARS-CoV-2 Alpha B.1.1.7 S1 and hACE2 (IC_50_ = 12.21) (Fig. 2B) and Delta B.1.617.2 S1 and hACE2 (IC_50_ = 15.33) (Fig. 2C). Conversely, the serum from intranasal immunization with S1 alone demonstrated poor inhibition of the interaction between SARS-CoV-2 Alpha B.1.1.7 S1 and hACE2 (IC_50_ = 0.98) (Fig. 2B) and Delta B.1.617.2 S1 and hACE2 (IC_50_ = 0.93) (Fig. 2C). These results suggest that TSLP can enhance neutralizing antibody production when administered by intranasal rather than intramuscular or intraperitoneal routes.

Next, we asked whether TSLP-induced neutralizing antibodies following intranasal vaccination may prevent SARS-CoV-2 pseudovirus infection using a pseudovirus neutralization titer assay (50% inhibitory dilution, PsVNT_50_). We found that S1 alone intranasal immunization produced low serum-neutralizing antibodies against SARS-CoV-2 WT (Fig. 2D), Alpha B.1.1.7 (Fig. 2E), and Delta B.1.617.2 (Fig. 2F) pseudoviruses, with PsVNT_50_ values of 1.01, 1.89, and 1.33, respectively. However, intranasal vaccination with S1 in the presence of TSLP elicited readily detectable serum-neutralizing antibodies against SARS-CoV-2 WT (Fig. 2D), Alpha B.1.1.7 (Fig. 2E), and Delta B.1.617.2 (Fig. 2F) pseudoviruses, with PsVNT_50_ values of 27.70, 22.04, and 19.91, respectively, which were approximately 27-fold, 12-fold, and 15-fold greater compared with S1 alone immunization. Overall, our data suggest that intranasal immunization with TSLP can effectively enhance the production of vaccine-induced neutralizing antibodies against SARS-CoV-2 WT and variant strains.

### TSLP induces DC activation and migration following intranasal immunization

**Fig 3:**
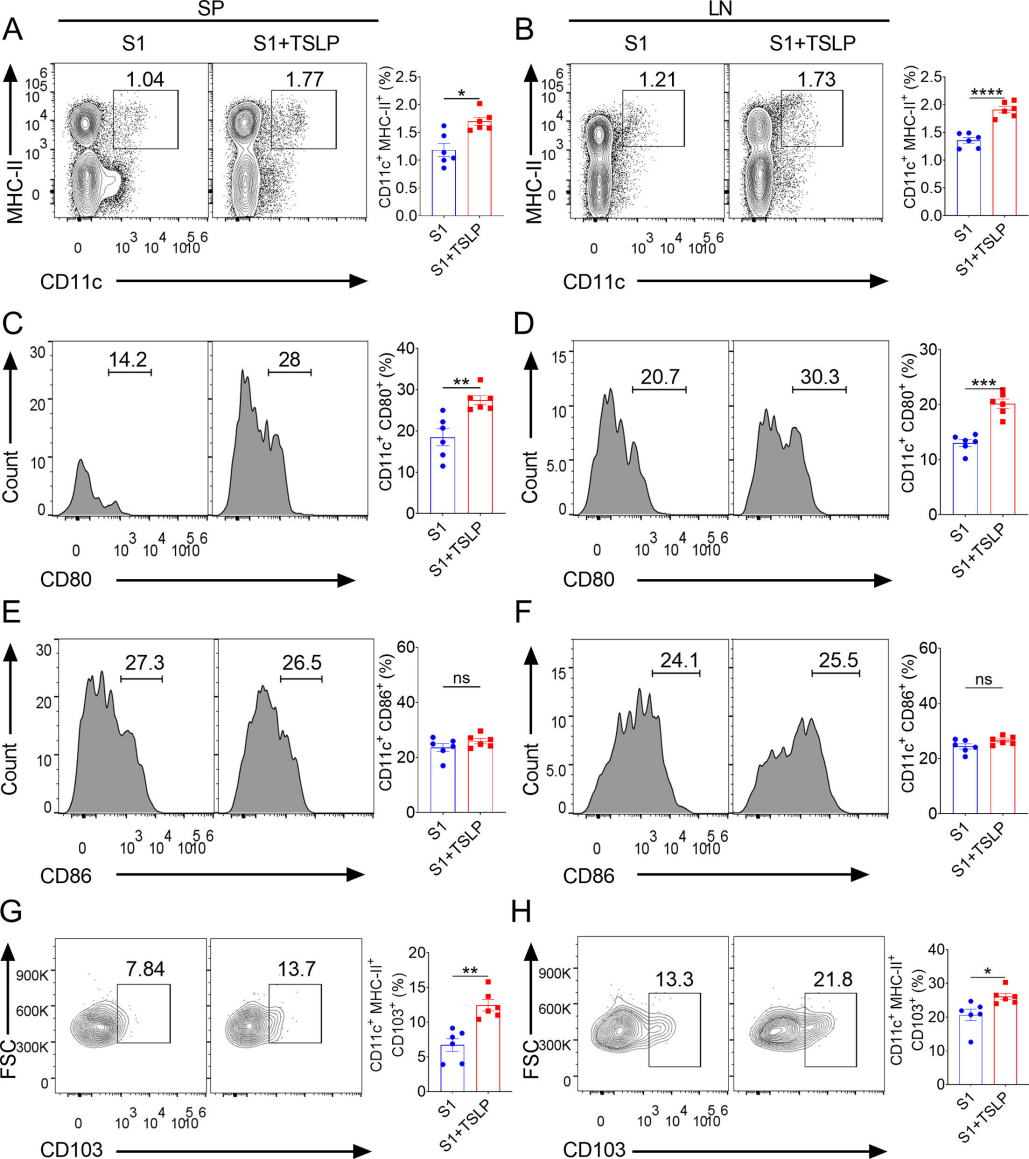
TSLP promotes DC activation and migration after intranasal immunization. WT mice (*n* = 6 mice per group) were intranasally immunized with SARS-CoV-2 S1 (2 µg) in the presence or absence of TSLP (2 µg). Booster immunizations were performed on 10 days and 20 days. Five days after the second booster immunization, the spleen (SP) and draining lymph nodes (LN) were collected. The percentages of CD11c^+^ MHC-II^+^ DCs (**A-B**), CD80^+^ cells among live CD11c^+^ cells (**C-D**), and CD86^+^ cells among live CD11c^+^ cells (**E-F**), and CD103^+^ cells among live CD11c^+^ MHC-II^+^ cells (**G-H**) in SP and LN were detected by FACS. Data are shown as mean ± SEM, *P < 0.05, **P < 0.01, ***P < 0.001, ****P < 0.0001, ns, no significant difference, by unpaired two-tailed Student’s *t*-test.

Dendritic cells (DCs) act as a bridge between antigen presentation and adaptive immune responses (27, 28). Maturation of DCs expresses CD11c, and they have to modulate to high surface molecule major histocompatibility complex (MHC) class II (MHC-II) and costimulatory molecules such as CD80 and CD86, which is an essential process for activating antigen-specific adaptive immunity (27, 28). We therefore sought to investigate whether TSLP affects DC maturation and activation during intranasal immunization. On day 5, after two booster intranasal immunizations, the DC surface markers CD11c and MHC-II were used to determine the population of mature DCs using flow cytometry. Our findings indicate that the TSLP-adjuvant S1 vaccination significantly increased the frequency of mature DCs (CD11c^+^ MHC-II^+^) in the spleen (*P* < 0.05) and draining lymph nodes (*P* < 0.001) compared with mice immunized with the S1 vaccine alone (Fig. 3A and B). Meanwhile, the CD11c^+^ CD80^+^ mature or activated DC populations in the spleen (*P* < 0.01) and draining lymph nodes (*P* < 0.001) of the TSLP plus S1 vaccination group were significantly higher than those in the S1 alone vaccination group (Fig. 3C and D), although CD11c^+^ CD86^+^ DC populations did not increase significantly (Fig. 3E and F). Upon maturation and activation, DCs migrate from the periphery to draining lymph nodes, which is a key step in inducing vaccine-mediated adaptive immunity (29). Since TSLP enhances the maturation and activation status of DCs during intranasal immunization, we evaluated whether this would also enhance DC migration. We observed a higher proportion of migratory DCs (CD103^+^ CD11c^+^ MHC-II^+^) in the spleen (*P* < 0.01) and draining lymph nodes (*P* < 0.05) of the TSLP-adjuvanted S1 vaccination group compared with the S1 alone vaccination group (Fig. 3G and H). Taken together, these results indicated that TSLP can promote DC activation and migration after intranasal immunization.

### TSLP boosts GC responses after intranasal immunization

**Fig 4:**
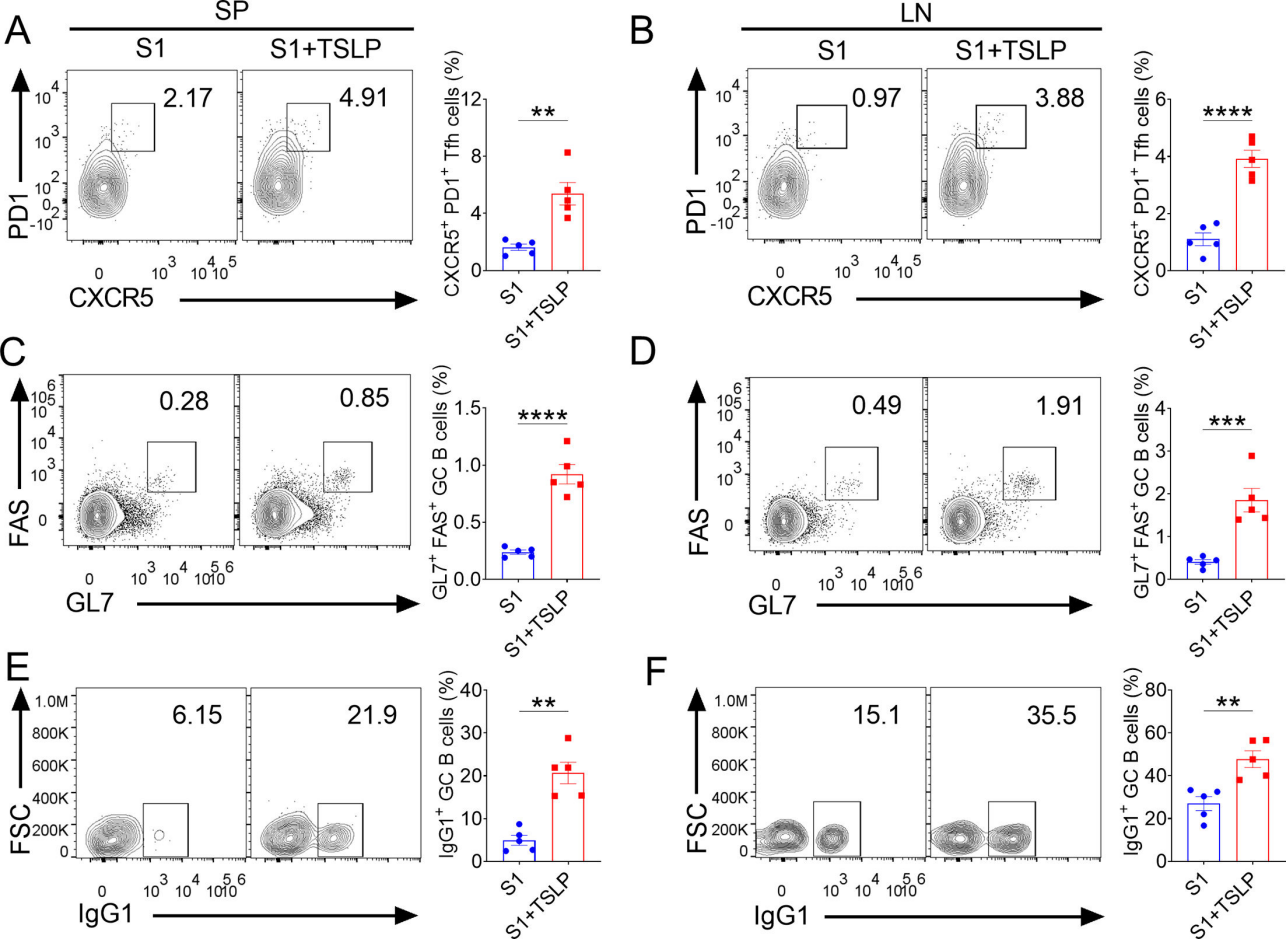
TSLP enhances Tfh cells and GC B cell responses after intranasal immunization. WT mice (*n* = 5 mice per group) were immunized by intranasal application of SARS-CoV-2 S1 (2 µg) alone or in combination with TSLP (2 µg). Booster immunizations were performed on 10 and 20 days. Ten days after the second booster immunization, the spleen (SP) and draining lymph nodes (LN) were harvested. The percentages of CXCR5^+^ PD-1^+^ Tfh cells among live CD19^-^ CD4^+^ CD44^+^ cells (**A-B**), Fas^+^ GL7^+^ GC B cells among live CD4^-^ CD19^+^ cells (**C-D**), and IgG1^+^ GC B cells among live CD4^-^ CD19^+^ Fas^+^ GL7^+^ cells (**E-F**) in the SP and LN were analyzed by flow cytometry. Data are shown as mean ± SEM, **P < 0.01, ***P < 0.001, ****P < 0.0001, by unpaired two-tailed Student’s *t*-test.

DCs can effectively promote germinal center (GC) reactions, whereas T follicular helper (Tfh) cells and B cells in GC are required for high-affinity antibody synthesis (29–31). We next used flow cytometry to determine the frequencies of Tfh cells (defined as live CD19^-^ CD4^+^ CD44^+^ PD1^+^ CXCR5^+^ T cells) and GC B cells (defined as live CD4^-^ CD19^+^ Fas^+^ GL7^+^ B cells) in the spleen and draining lymph nodes of WT mice inoculated with S1 or TSLP-supplemented S1 vaccine by intranasal immunization. We found that intranasal immunization with a TSLP-enriched vaccine increased the proportion of PD1^+^ CXCR5^+^ Tfh cells among the live CD19^-^ CD4^+^ CD44^+^ cells in the spleen (*P* < 0.01) and lymph nodes (*P* < 0.0001) when compared with S1 immunization alone (Fig. 4A and B). Similarly, the proportion of GL7^+^ FAS^+^ GC B cells among the live CD4^-^ CD19^+^ cells in the spleen (*P* < 0.0001) and lymph nodes (*P* < 0.001) of WT mice immunized with TSLP combined with S1 was considerably increased compared with S1 immunization alone (Fig. 4C and D). Furthermore, WT mice immunized with TSLP-adjuvanted vaccine had a higher frequency of IgG1-producing GC B cells (live CD4^-^ CD19^+^ GL7^+^ FAS^+^ IgG1^+^) in the spleen (*P* < 0.01) and lymph nodes (*P* < 0.01) compared with WT mice immunized with S1 alone (Fig. 4E and F). However, intranasal treatment of WT mice with TSLP alone did not result in such GC reactions (Fig. S2). These findings suggest that TSLP promotes Tfh cell and GC B cell responses after intranasal immunization.

### TSLP enhances mucosal adaptive immunity by directly targeting DCs

**Fig 5:**
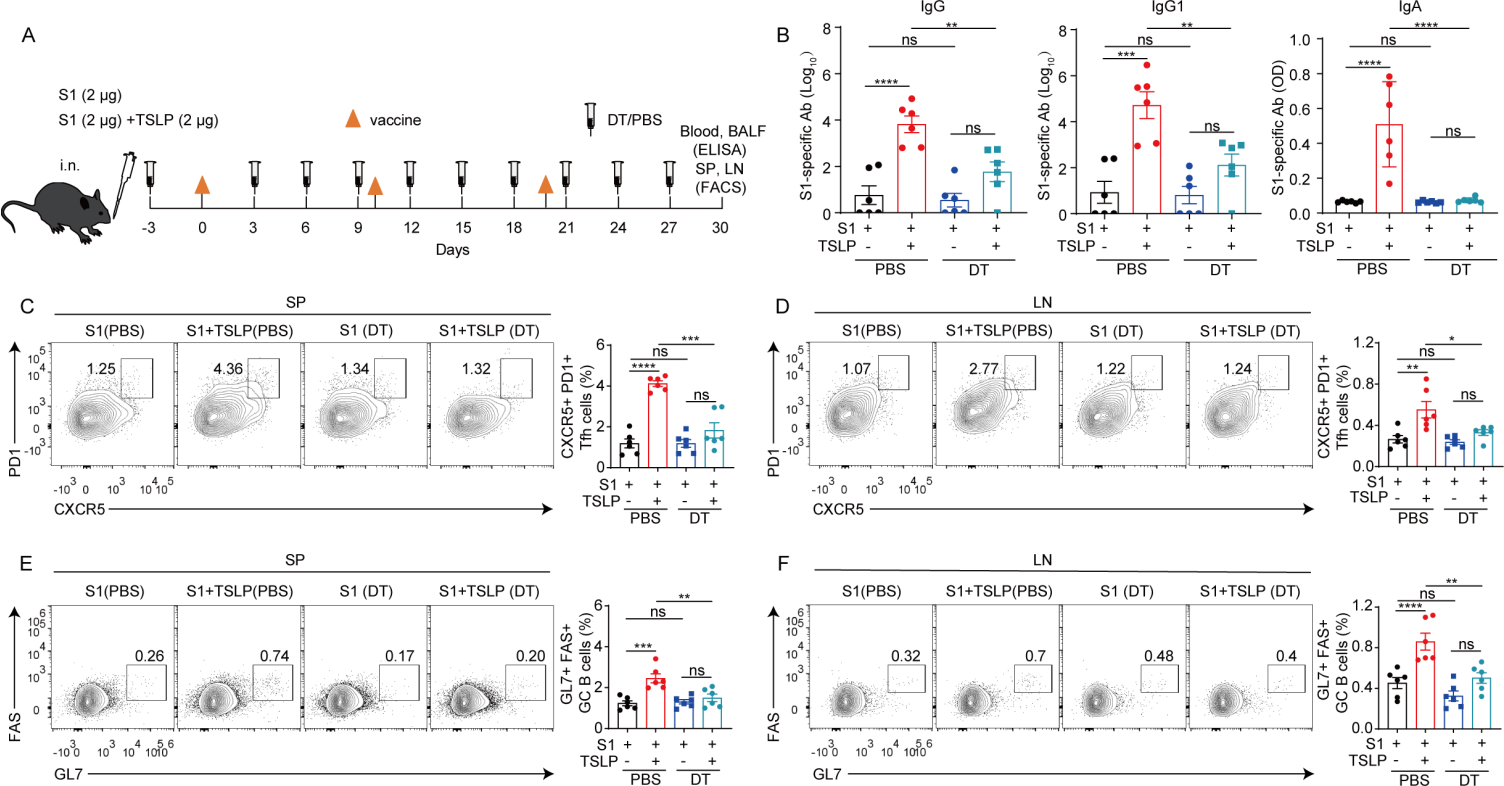
DC is required for TSLP-induced adaptive responses after intranasal vaccination. (**A**) CD11c-DTR mice (*n* = 6) underwent intranasal vaccination with SARS-CoV-2 S1 (2 µg) alone or a combination of SARS-CoV-2 S1 (2 µg) and TSLP (2 µg). Booster immunizations were performed 10 and 20 days later. S1 and S1 + TSLP immunized were injected with either DT (diphtheria toxin) or PBS every 3 days. Serum, BALF, spleen (SP), and draining lymph nodes (LN) were collected on day 30. (**B**) Serum S1-specific IgG and IgG1 and BALF S1-specific IgA titers were detected by ELISA. The frequencies of CXCR5^+^ PD-1^+^ Tfh cells among live CD19^-^ CD4^+^ CD44^+^ cells (**C-D**) and Fas^+^ GL7^+^ GC B cells among live CD4^-^ CD19^+^ cells (**E-F**) in SP and LN were detected by FACS. Data are shown as mean ± SEM, *P < 0.05, **P < 0.01, ***P < 0.001, ****P < 0.0001, ns, no significant difference, by two-way ANOVA with Dunnett’s multiple-comparison test.

To investigate whether the adaptive response enhanced by TSLP is dependent on DCs, we performed similar vaccination protocols in CD11c-DTR (Diphtheria Toxin Receptor) mice (Fig. 5A). After PBS or diphtheria toxin (DT) treatment, CD11c-DTR mice with or without CD11c^+^ DC depletion were intranasally immunized with S1 in the presence or absence of TSLP, and vaccine-mediated antibody levels and GC responses were examined by ELISA and flow cytometry on day 10 after two booster immunizations (Fig. 5A). When CD11c-DTR mice received S1 vaccination alone, there was no difference in serum S1-specific IgG and IgG1 and BALF S1-specific IgA production between DT and PBS treatment (Fig. 5B). However, in the TSLP-adjuvant S1 vaccine-immunized CD11c-DTR mice, DT treatment resulted in substantially lower levels of serum S1-specific IgG (*P* < 0.01) and IgG1 (*P* < 0.01) and BALF S1-specific IgA (*P* < 0.0001) compared with PBS treatment (Fig. 5B). In line with the decreased antibody response, DT treatment decreased the frequency of Tfh cells (Fig. 5C and D) and GC B cells (Fig. 5E and F) in the spleen and lymph nodes of TSLP-adjuvant S1 vaccine-immunized CD11c-DTR mice in comparison to PBS treatment.

**Fig 6:**
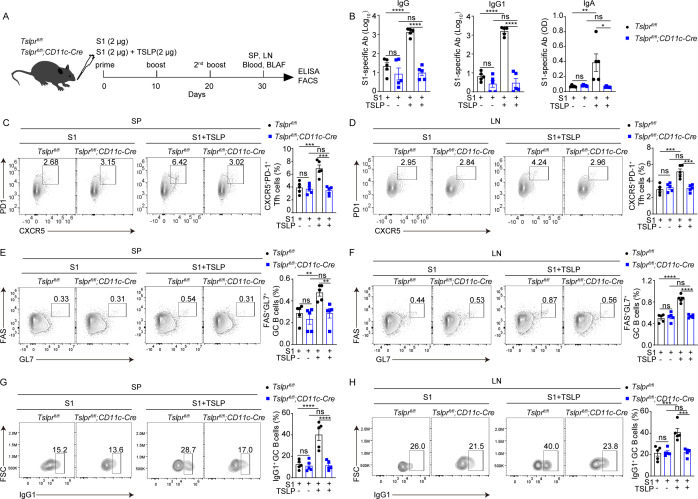
TSLP receptor deficiency on DCs impairs vaccine-induced adaptive responses. (**A**) *Tslpr^fl/fl^* (*n* = 5) and *Tslpr^fl/fl^*; *CD11c*-Cre mice (*n* = 5) were intranasally immunized with SARS-CoV-2 S1 (2 µg) in the presence or absence of TSLP (2 µg). Booster immunizations were performed 10 and 20 days later. Serum, BALF, the spleen (SP), and draining lymph nodes (LN) samples were collected on day 10 after the second booster immunization. (**B**) Serum S1-specific IgG and IgG1 and BALF S1-specific IgA titers were determined by ELISA. The percentages of CXCR5^+^ PD-1^+^ Tfh cells among live CD19^-^ CD4^+^ CD44^+^ cells (**C-D**), Fas^+^ GL7^+^ GC B cells among live CD4^-^ CD19^+^ cells (**E-F**), and IgG1^+^ GC B cells among live CD4^-^ CD19^+^ Fas^+^ GL7^+^ cells (**G-H**) in SP and LN were measured by FACS. Data are shown as mean ± SEM, *P < 0.05, **P < 0.01, ****P < 0.0001, ns, no significant difference, by two-way ANOVA with Dunnett’s multiple-comparison test.

To further confirm whether TSLP signaling on DC is required for inducing mucosal adaptive immunity, we mated conditional *CD11c*-Cre mice with floxed TSLPR (*Tslpr^fl/fl^*) mice to obtain *Tslpr^fl/fl^; CD11c*-Cre mice, which were utilized to specifically delete TSLPR on CD11c^+^ DCs. When these animals were intranasally immunized with the S1 vaccine formulated with TSLP (Fig. 6A), the levels of S1-specific serum IgG (*P* < 0.0001) and IgG1 (*P* < 0.0001) and BALF IgA (*P* < 0.05) in *Tslpr^fl/fl^; CD11c-*Cre mice were consistently lower than in *Tslpr^fl/fl^* mice on day 10 after two booster intranasal vaccinations (Fig. 6B). However, *Tslpr^fl/fl^* mice and *Tslpr^fl/fl^; CD11c-*Cre mice showed no difference in S1-specific serum IgG and IgG1 and mucosal IgA levels following intranasal immunization with S1 alone (Fig. 6B). Furthermore, we found that *Tslpr^fl/fl^; CD11c-*Cre mice had a decreased proportion of Tfh cells, GC B cells, and IgG1-producing GC B cells in the spleen (Fig. 6C, E, and G) and lymph nodes (Fig. 6D, F, and H) on day 10 after two booster intranasal immunizations with the TSLP-formulated S1 vaccine compared with *Tslpr^fl/fl^* mice, demonstrating that TSLP induces mucosal adaptive immune responses in a DC-dependent manner.

### The TSLP-adjuvanted vaccine confers protection against SARS-CoV-2 WT and Delta strains

**Fig 7:**
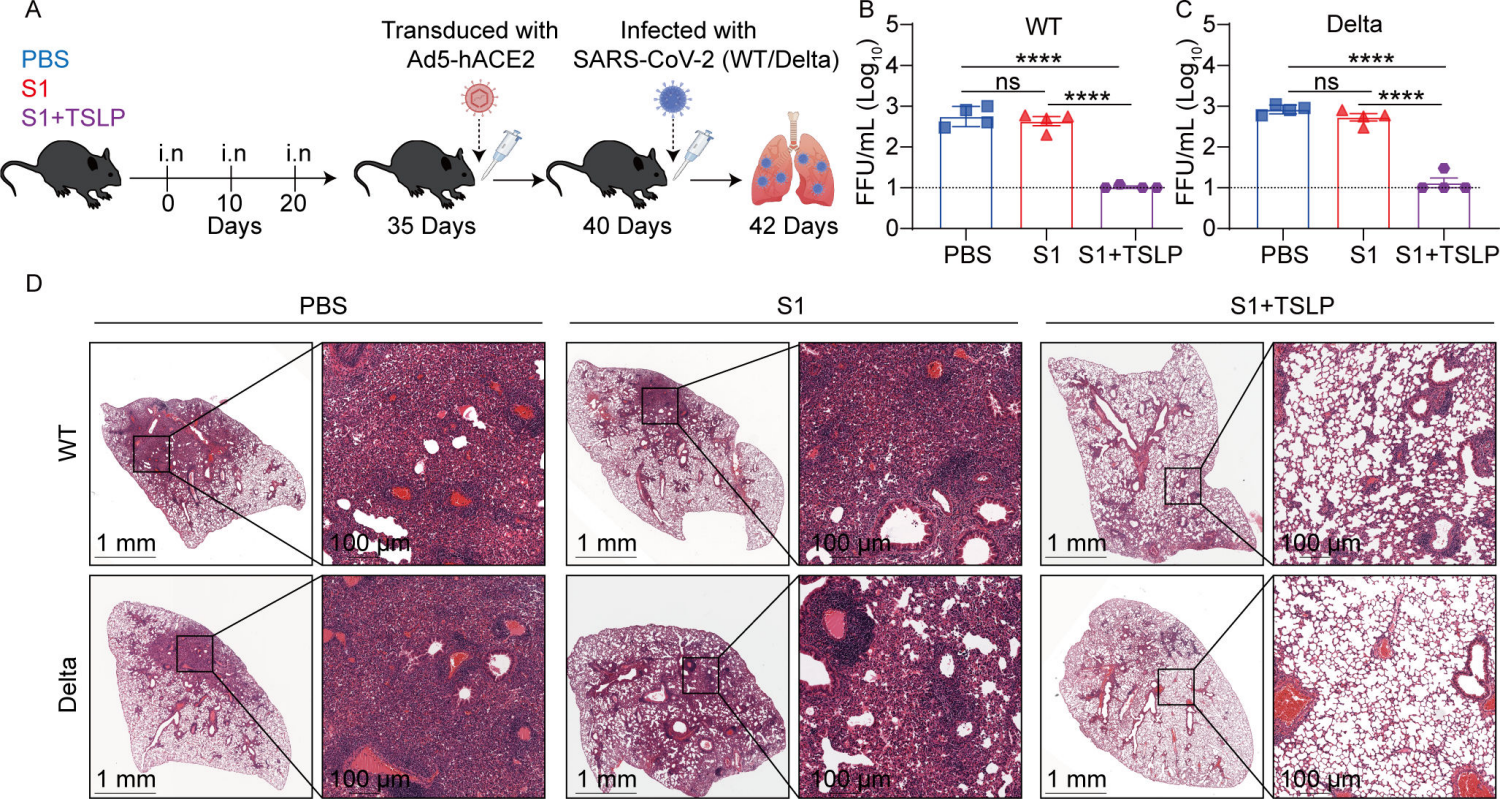
The TSLP-adjuvanted subunit vaccine provides cross-protection against SARS-CoV-2. (**A**) Schematic diagram of mouse viral challenge. WT mice (*n* = 4 per group) were intranasally given PBS, SARS-CoV-2 S1 (2 µg), or SARS-CoV-2 S1 (2 µg) + TSLP (2 µg) on days 0, 10, and 20. On day 35, mice were transduced with 2.5 × 10^8^ FFU of Ad5 expressing hACE2 and then intranasally challenged with 5 × 10^4^ FFU SARS-CoV-2 wild-type (WT) or 5 × 10^4^ FFU B.1.617 (Delta) strains on day 40, and mice were sacrificed on day 42. Virus titrations in lungs were detected (**B-C**), and the lung tissues were harvested for H&E staining (**D**). Data are shown as mean ± SEM. ***P < 0.001, ns, no significant difference, by unpaired two-tailed Student’s *t*-test.

To ascertain the biological significance of TSLP in enhancing vaccine-induced immune responses, WT mice were immunized intranasally with S1 three times at 10-day intervals, either with or without TSLP. PBS was administered to control mice under the same settings (Fig. 7A). Following the transduction of immunized and unimmunized mice with 2.5 × 10^8^ FFU of Ad5-expressing hACE2, they were then challenged intranasally with either 5 × 10^4^ FFU of the SARS-CoV-2 wild-type (WT) or the B.1.617.2 strain (Delta). This process facilitated ectopic expression of hACE2, enabling effective infection by SARS-CoV-2 (32). Two days after virus infection, the lung tissues of mice were tested for viral titer and stained with HE (Fig. 7A). We observed no difference in viral titer (Fig. 7B and C) and pathological damage (Fig. 7D) in the lungs of mice immunized with S1 alone compared with mice treated with PBS. However, mice administered the TSLP-supplemented S1 vaccine showed a lower viral load following SARS-CoV-2 WT (*P* < 0.0001) or Delta (*P* < 0.0001) strain infection than mice administered S1 alone (Fig. 7B and C). Furthermore, histological analysis of the lung pathological damage revealed that compared with mice immunized with the S1 vaccine alone, lung damage in mice immunized with S1 supplemented with TSLP was reduced after SARS-CoV-2 WT or Delta strain infection (Fig. 7D). These findings suggest that mucosal vaccines adjuvanted with TSLP can protect mice against a broad spectrum of SARS-CoV-2 WT and Delta strains.

## DISCUSSION

The present research investigated how distinct immunization routes impact the regulation of TSLP on SARS-CoV-2 subunit vaccine-mediated humoral responses. Our findings indicate that TSLP can promote the generation of SARS-CoV-2 vaccine-specific IgG and IgG1 antibodies through mucosal and non-mucosal immunization routes, whereas it can only enhance mucosal IgA and high-affinity neutralizing antibody responses by intranasal immunization. The mucosal adjuvant effect of TSLP hinges on directly targeting DC to induce activation and migration, hence enhancing Tfh and GC B cell responses. Intranasal vaccination with the SARS-CoV-2 subunit vaccine containing TSLP provides effective protection against SARS-CoV-2 WT and Delta strains. Therefore, our data suggest that mucosal immunization represents the most effective way for TSLP to exert adjuvant activity, efficiently inducing both systemic and mucosal adaptive immune responses.

TSLP was recently identified as a new adjuvant (18–20). Our previous studies demonstrated that TSLP exhibits adjuvant activity on influenza subunit vaccines when immunized by rectal routes and improves the efficacy of rectally administered influenza vaccines (21). TSLP signaling is also required for interferon-λ-induced mucosal adaptive immunity after being intranasally immunized with influenza vaccines, highlighting its importance as a mucosal adjuvant (19, 20, 22). However, the most appropriate route of administration and mechanism of action for TSLP to exert its adjuvant activity during subunit vaccine immunization remains unclear. Here, we investigated the effect of TSLP on humoral immunity when the SARS-CoV-2 subunit vaccine was administered via multiple conventional routes, including intranasal, intramuscular, and intraperitoneal immunization. Our results showed that TSLP boosts vaccine-specific IgG and IgG1 but not IgG2c antibody generation through three immune sites, which is consistent with previous reports that TSLP acts as a mucosal adjuvant that shifts HIV gp140 antigen-specific IgG synthesis toward IgG1 without affecting IgG2c production (18). Mice lacking TSLPR signaling exhibit low levels of serum titer of antigen-specific IgG1 antibodies after intraperitoneal or cutaneous immunization but show no influence on IgG2c production (14, 33). TSLP is widely recognized as an inducer of T helper (Th) 2 immunity (8, 16). TSLP promotes the production of Th2-related cytokines (such as interleukin-4, IL-4) but has no effect on the production of Th1-related cytokines (such as interferon-γ, IFN-γ) during mucosal vaccination (18). Loss of TSLP signaling impairs Th2-associated cytokines IL-4, IL-5, and IL-13 production but does not affect Th1-associated cytokine production (34, 35). IL-4 promotes IgG1 synthesis, whereas IFN-γ supports IgG2c generation (36, 37). However, whether TSLP-induced isotype class switching toward IgG1, by promoting Th2-related cytokine production, needs further investigation. As a result, these findings suggest that TSLP exhibits a broad adjuvant profile and enhances IgG1-biased immune responses.

Surprisingly, in this research, only intranasal vaccination with TSLP produced robust mucosal IgA and neutralizing antibody production. These results are consistent with previous reports indicating that intranasal vaccination with TSLP boosts vaccine-specific IgA synthesis in vaginal lavage and that intranasal immunization elicited stronger vaccine-specific IgA responses than non-mucosal immunization when the vaccine was delivered with TSLP (18). These findings provide credence to the hypothesis that mucosal immunization may be the most efficient approach for TSLP to exert adjuvant activity. Most current subunit vaccines are delivered intramuscularly or subcutaneously, which frequently fails to produce sufficient mucosal protection (6, 38, 39). Therefore, the mucosal adjuvant features of TSLP could contribute to addressing the inadequacies of current subunit vaccines. Considering that mucosal immunization causes less discomfort and more compliance, mucosal delivery of TSLP-containing vaccines may be beneficial for vaccine immunization in children and the elderly (2, 40, 41).

GC responses are critical for vaccine-induced adaptive immunity, and Tfh cells coordinate GC formation, assisting antigen-specific GC B cells to produce high-affinity antibodies (14, 30, 31). Our previous studies showed that TSLP signaling enhances influenza vaccine-mediated Tfh and GC B cell responses following mucosal immunization and is required for IFN-λ-boosted Tfh and GC B cell responses (19–22). In agreement with these conclusions, we determined that TSLP promotes Tfh and GC B cell responses when the SARS-CoV-2 subunit vaccine is delivered intranasally. The enhancement effect of TSLP on GC responses and SARS-CoV-2 subunit vaccine-specific IgG1 generation was achieved by acting directly on DCs. It is well known that DCs provide three signals to support Tfh cell activation and differentiation, including peptide-MHC-II, multiple costimulatory molecules, and cytokines (27). Our results showed that TSLP mainly promoted DC activation by increasing MHC-II expression and inducing high levels of costimulatory molecule CD80 molecules after intranasal immunization, which are crucial for Tfh cell activation and differentiation (42). Although we do not yet understand the molecular mechanism by which TSLP-activated DCs induce Tfh cell differentiation to encourage B cells to preferentially produce IgG1 in our mouse model, TSLP signaling on DCs drives Tfh cells to secrete IL-4 in a GATA3-dependent manner, which promotes IgG1^+^ GC B cell and IgE^+^ GC B cell generation (33, 43). Moreover, TSLP-induced human Tfh cell differentiation by activating DCs expressing the costimulatory molecule OX40L, which stimulates antibody secretion by B cells in an IL-4Rα-dependent manner (15). However, whether and how TSLP-activated DC cells polarize Tfh cells via the IL-4 signaling pathway to induce GC B cell responses, resulting in IgG1-biased immune responses after intranasal administration of the SARS-CoV-2 subunit vaccine, deserves further exploration. It should be underscored that IL-4 is a master regulator of Th2 immunity, and it was previously considered to be produced by Th2 cells that help B cells in triggering the IgG1 and IgE class switching (37). In this work, it cannot be ruled out that the activation of TSLP-induced DCs triggers the IgG1 production by simultaneously promoting Tfh cell and Th2 cell immune responses in an IL-4 signaling pathway-dependent manner. Collectively, these studies indicate that TSLP may induce Tfh differentiation by stimulating DC activation, thereby driving GC B cell responses.

Several DC subtypes can initiate Tfh cell differentiation, but migratory DCs have been proposed as the dominant Tfh cell-priming DC subset after vaccination (29). Our previous findings showed that during intranasal influenza vaccine administration, IFN-λ elicited a germinal center reaction by inducing respiratory microfold cells to secrete TSLP and promoting DC migration to lymph nodes (19, 20). The absence of migration of DCs impairs TSLP-induced Tfh and GC B cell responses and IgG1 production (19). In this study, we unanimously observed that TSLP expands the number of CD103^+^ DCs in draining lymph nodes, which might enable DCs to transport antigens and trigger Tfh and GC B cell responses. Taken together, these findings indicate that TSLP targets DCs to induce activation and migration, which may be vital for its mucosal adaptive immunity-enhancing effects.

TSLP and IL-7 possess a common IL-7Rα chain, indicating a close connection (9). IL-7 is known to play a vital role in the development of T cells, B cells, certain subsets of NK cells, and dendritic cells (DCs) (9). However, the effect of IL-7 on Tfh cell differentiation remains debated (44–46). IL-7 can induce Tfh cell activation and differentiation, leading to the activation and expansion of GC B cells (44, 45). Other studies found contradicting data demonstrating that IL-7 signaling may repress Tfh-associated genes, including Bcl6 and CXCR5, in a STAT5-dependent manner, indicating an inhibitory role of IL-7 in Tfh cell differentiation (46). Interestingly, IL-7-boosted Tfh and GC B cell reactions induce both influenza vaccine-specific IgG1 and IgG2c production (45), which conflicts with our finding that TSLP preferentially induces IgG1 rather than IgG2c responses. Mechanistically, our study clearly revealed that TSLPR signaling on DCs is required for TSLP-enhanced GC responses to induce IgG1-biased humoral immunity. Since both CD4^+^ T cells and DCs express IL-7R, implying that IL-7 might act directly on CD4^+^ T cells or indirectly on DCs to regulate GC responses (9), probably leading to inconsistent adaptive immunity regulation properties of TSLP.

Vaccine-induced antibody response plays a crucial role in combating SARS-CoV-2 infections (6, 23, 24). The development of SARS-CoV-2 variants, such as Alpha B.1.1.7 and Delta B.1.617.2, has promoted concerns that these variants may escape neutralizing antibody responses induced by current vaccines (47, 48). Interestingly, our results indicate intranasal immunization with the SARS-CoV-2 enriched with TSLP robustly induces neutralizing antibodies to SARS-CoV-2 wild-type, Alpha B.1.1.7, and Delta B.1.617.2 pseudoviruses. Furthermore, TSLP-induced mucosal adaptive immunity provides cross-protection against SARS-CoV-2 wild-type and Delta B.1.617.2 strains, indicating that TSLP can boost vaccine-induced antiviral immunity.

Overall, our work highlights the adjuvant effect of TSLP in enhancing systemic and mucosal immune responses to the SARS-CoV-2 subunit vaccine through the mucosal immune route. We found that TSLP exhibits an immune-enhancing effect on adaptive immunity by directly stimulating DC activation and migration, which boosts germinal center reactions. Vaccine supplementation with TSLP effectively protects against SARS-CoV-2 and its variants. Therefore, the adjuvant activity of TSLP should be considered when developing mucosal vaccines and improving vaccination efficacy in humans.

## MATERIALS AND METHODS

### Mice

Female C57BL/6 mice (designated wild-type mice, WT mice) at 6–8 weeks were obtained from Guangdong Medical Laboratory Animal Centre (Guangzhou, China). *CD11c*-DTR mice were gifted by Professor Fei Li (Fudan University, Shanghai, China).

To generate dendritic cell-specific *Tslpr* knockout mice, *CD11c*-Cre mice were procured from Janvier Laboratories, whereas *Tslpr^fl/fl^* mice were obtained from GemPharmactech (China). Dendritic cell-specific *Tslpr* knockout mice (designated as *Tslpr^fl/fl^*; *CD11c*-Cre) were produced by crossing *CD11c*-Cre mice with *Tslpr^fl/fl^* mice and confirmed through tail genotyping. *Tslpr^fl/fl^* mice were used as the control group.

All mice (female, 6–8 weeks) used in this work were maintained under specific pathogen-free (SPF) conditions in the local animal facility.

### Immunizations

For intranasal immunizations (i.n.), WT mice, *Tslpr^fl/fl^* mice, and *Tslpr^fl/fl^*; *CD11c*-Cre mice were anesthetized with isoflurane and then immunized intranasally with either SARS-CoV-2 wild-type (Wuhan) S1 (designated S1, 2 µg) alone (Z03501, Genscript) or a combination of S1 (2 µg) and mouse TSLP (2 µg) (CJ69, Novoprotein) in a total volume of 30 µL. Booster immunizations were performed 10 and 20 days later, respectively. On the 10th day after the second booster immunization, serum, and bronchoalveolar lavage fluid (BALF) samples were collected, and S1- or RBD-specific antibody responses were assessed by ELISA. Some serum samples were used for the SARS-CoV-2-hACE2 competition assay and pseudovirus neutralization assays. The spleen and draining lymph nodes were collected on day 5 or 10 after the second booster immunization for flow cytometry assays.

For intramuscular immunization (i.m.), WT mice were injected intramuscularly with the S1 (2 µg) vaccine alone or a combination of the S1 (2 µg) and TSLP (2 µg) vaccines in a total of 100 µL volume on days 0 and 10. On the 10th day after the booster immunization, serum and BALF were obtained, and S1- or RBD-specific antibody levels were assessed by ELISA.

For intraperitoneal immunization (i.p.), WT mice were administered intraperitoneally with S1 (2 µg) vaccine alone or a combination of S1 (2 µg) and TSLP (2 µg) vaccines in a total of 200 µL volume, followed by booster immunizations 10 days later. On the 10th day after the last immunization, serum and BALF samples were collected, and S1- or RBD-specific antibody responses were assessed by ELISA.

For the immunization of CD11c DTR mice, diphtheria toxin (DT, Sigma-Aldrich) at a dose of 1.5 ng/g body weight was administered via intraperitoneal injection on days −3, 3, 6, 9, 12, 15, 18, 21, 24, and 27 to deplete dendritic cells (DCs) *in vivo*. Control mice were given an equal volume of PBS. At days 0, 10, and 20, the animals were anesthetized with isoflurane and intranasally immunized with 2 µg of S1 protein in the presence or absence of 2 µg of TSLP. On day 10 after the second booster immunization, serum and BALF were collected for ELISA analysis of S1-specific antibodies, whereas spleens and draining lymph nodes were harvested for FACS analysis of T follicular helper (Tfh) cells and germinal center (GC) B cells.

### Serum collection

Blood was collected from all mice 10 days after one or two booster immunizations via the retro-orbital plexus. Blood samples were allowed to clot at room temperature for 1 h and then centrifuged at 12,000 rpm for 5 min. The serum was carefully transferred into new microcentrifuge tubes while avoiding any clots or cellular debris. The serum samples were then stored at −80°C for further examination.

### Preparation of bronchoalveolar lavage fluid (BALF)

Bronchoalveolar lavage fluid (BALF) was collected by instilling 500 µL of PBS into the lungs through the trachea using a peripheral venous catheter (BD Venflon) and lavaging three times. BALF was centrifuged at 2,000 rpm for 4 min, and the supernatants were kept at −80°C before being analyzed for IgA levels.

### Measuring S1- or RBD-specific antibodies

To detect SARS-CoV-2 S1 or RBD-specific antibodies in serum and BALF, ELISAs were performed using a 96-well plate (446469, Thermo Fisher). The plate was coated with either SARS-CoV-2 WT S1 (Z03501, Genscript) or RBD (Z03483, Genscript) and incubated at 4°C overnight. After coating, the plate was blocked using a 5% skim milk solution for 1 h at 37°C before being rinsed with PBST (PBS buffer with 0.05% Tween 20). Serially diluted serum or undiluted BALF was incubated for 1 h at 37°C. Following incubation, the plate was washed again with PBST three times. The binding antibodies were then detected using a horseradish peroxidase (HRP)-conjugated goat anti-mouse IgG antibody (62–6520, Invitrogen), IgG1 (A10551, Invitrogen), IgG2c (ab97255, abcam), and IgA (626720, Invitrogen). The bound antibodies were visualized by incubating the plate with a tetramethylbenzidine (TMB) substrate reagent kit (H101-01, Transgen) for 10–30 min. The reaction was then terminated by adding 1 M HCl, and the absorbance was measured at 450  nm using a SpectraMax 340PC Microplate Reader (Molecular Devices, USA). ELISA endpoint titers were determined as the greatest dilutions that resulted in twice greater (S1- or RBD-specific IgG subtypes) OD values than the background. Considering IgA titers in BALF were rather low, undiluted samples were used for ELISA, with OD values plotted directly.

### Competitive ELISA

A competitive ELISA was employed to evaluate the inhibitory effect of immunized serum on the binding of SARS-CoV-2 WT, Alpha, and Delta S1 to human angiotensin-converting enzyme (hACE2), respectively. Ninety-six microtiter plates were coated with 400 ng of recombinant hACE2 protein (T101301, East Mab) in a total of 50 µL volume and induced overnight at 4°C. After washing and blocking, the 400 ng S1 proteins (His Tag) of SARS-CoV-2 WT (Genscript, Z03485), Alpha B.1.1.7 (East Mab, China), and Delta B.1.617.2 (East Mab, China) were pre-incubated with 3-fold serially diluted serum (6-point dilutions starting at 1:2) before being added to the hACE2-coated plates. After a 1 h incubation at 37°C to allow for antibody binding, the bound antibodies were detected using an anti-His antibody (HRP) (HRP-66005, Proteintech). After washing with PBST, 50 µL of TMB substrate was added, followed by 50 µL of 1 M HCl to stop the reaction. The wells lacking S1 proteins were used as negative controls, and wells containing S1 proteins but lacking serum were used as positive controls. The absorbance at OD_450_ was measured with a SpectraMax 340PC Microplate Reader (Molecular Devices, USA). The percent inhibition was calculated using the following equation: Inhibition (%) = [1 − (sample OD_450_ – negative control OD_450_) / (positive control OD_450_ − negative control OD_450_)] × 100%. The 50% inhibitory concentration (IC_50_) was calculated by sigmoidal curve fitting with four-parameter nonlinear regression using GraphPad Prism v.8.0 software.

### SARS-CoV-2 pseudovirus neutralization assay

For the SARS-CoV-2 pseudovirus neutralization assay, 2 × 10^4^ hACE2-expressing HEK293T cells (RM02456, ABclonal) were inoculated in 96-well plates (Corning, 3596) and cultured with 100 µL Dulbecco’s modified Eagle’s medium DMEM (11995065, Gibco) supplemented with 10% fetal bovine serum (FBS, FS301-02, TransGen) and 1× penicillin-streptomycin (P/S, FG101-01, TransGen) overnight at 37°C in a humidified atmosphere containing 5% CO_2_. Immunized and unimmunized serum at an initial dilution of 1:2.5 was 4-fold diluted (spanning 1:2.5 to 1:640) with DMEM medium supplemented with 10% FBS and 1× *P*/S and then pre-incubated with GFP-Luciferase pseudoviruses in 96-well plates for 1 h, including SARS-CoV-2 Wuhan (WT) (GM-0220PV07, Genomeditech), Alpha B.1.1.7 (GM-0220PV33, Genomeditech), and Delta B.1.617.2 (GM-0220PV45, Genomeditech). After that, serum-pretreated pseudovirus was added to hACE2-expressing HEK293T cells in 96-well plates and cultured for 48 h. Cells lacking pseudovirus were used as blank controls, and cells containing pseudovirus but lacking serum were used as virus controls. The luciferase activity was performed using a single luciferase reporter assay kit (FR101, TransGen) according to the manufacturer’s instructions. The relative light units (RLU) were measured with a SpectraMax Gemini XPS Microplate Reader (Molecular Devices, USA). The inhibition of PsV infection of each serum sample was calculated using the following formula: Inhibition of PsV infection (%) = [1 − (sample RLU – blank control RLU) / (virus control RLU − blank control RLU)] × 100%. The 50% pseudovirus neutralization titer (PsVNT_50_) of each sample was calculated by sigmoidal curve fitting with four-parameter nonlinear regression using GraphPad Prism v.8.0 software.

### Flow cytometry

Spleen and mediastinal lymph nodes (mLN) were collected and prepared for a single-cell suspension. Briefly, single-cell suspensions were prepared by mechanical dissociation of the spleen and mLN with a 10 mL syringe plunger and 70 µm cell strainer (352350, Falcon) in PBS supplemented with 2% FBS (FS301-02, TransGen) and 2 mM EDTA. The red blood cells of the spleen samples were lysed with ACK buffer (0.15  mM NH_4_Cl, 10  mM KHCO_3_, 0.1  mM EDTA-Na_2_ in Milli-Q-H_2_O). Single-cell suspensions were collected and washed with cold PBS, then suspended in FACS buffer (1% BSA, 0.1% NaN_3_ in PBS) and treated with anti-mouse CD16/32 antibodies (93, Biolegend) for 20 min on ice to block non-specific antibody binding. Dead cells were stained with LIVE/DEAD fixable near-IR dead cell dye (Invitrogen) for 30 min on ice. For staining of DCs, Tfh cells, and GC B cells, cells were incubated with anti-mouse antibody mixtures, including anti-CD11c (N418, BioLegend), anti-CD103 (2E7, BioLegend), anti-MHC-II (M5/114.15.2, BioLegend), anti-CD80 (16–10A1, eBioscience), anti-CD86 (GL1, eBioscience), anti-CD4 (GK1.5, BioLegend), anti-CD19 (MB19-1, BioLegend), anti-PD1 (29F.1A12, BioLegend), anti-CXCR5 (L138D7, BioLegend), anti-CD44 (IM7, BioLegend), anti-FAS (SA367H8, BioLegend), and anti-GL7 (GL7, BioLegend). For intracellular staining, cells were fixed and permeabilized with Cytofix/Cytoperm (554714, BD Biosciences) according to the manufacturer’s instructions before being treated with anti-IgG1 antibody (RMG1-1, BioLegend) for 45 min at room temperature. Cells were rinsed with FACS buffer and fixed with 1% paraformaldehyde (PFA) for 30 min prior to acquisition. All samples were run with the Attune NxT flow cytometer (Invitrogen, USA), and subsequent data analysis was performed with FlowJo software (Treestar).

DC subpopulation gating strategy: cells were pregated based on forward and side scatters, single cells, and live cells, and then, DCs were gated based on CD11c and MHC-II expression. CD11c^+^ CD80^+^ DCs were gated based on CD11c and CD80 expression. CD11c^+^ CD86^+^ DCs were gated based on CD11c and CD86 expression. Migratory CD103^+^ DCs were gated based on CD11c, MHC-II, and CD103 expression.

Tfh cells gating strategy: cells were pregated based on forward and side scatters, single cells, live cells, CD19^-^ cells, and then CXCR5^+^ PD-1^+^ Tfh cells were gated based on CD4, CD44, CXCR5, and PD-1 expression.

GC B cells gating strategy: cells were pregated by forward and side scatters, single cells, live cells, and CD4^-^ cells, and then Fas^+^ GL7^+^ GC B cells were gated based on CD19, Fas, and GL7 expression. IgG1^+^ GC B cells were gated based on CD19, Fas, GL7, and IgG1 expression.

### SARS-CoV-2 challenge

WT mice at 8 weeks were given intranasally 30 µL of 2 µg SARS-CoV-2 S1 protein in the presence or absence of 2 µg of TSLP, three times at 10-day intervals under isoflurane. The control mice were administered PBS under identical settings. The animals were transduced with 2.5 × 10^8^ FFU of adenoviral vectors expressing hACE2 (Ad5-hACE2) under the control of the CMV promoter. The overexpression of hACE2 *in vivo* was verified and beneficial to SARS-CoV-2 infection (32). Mice were intranasally infected with 5 × 10^4^ FFU of the SARS-CoV-2 (WT) strain (isolated from COVID-19 patients in Guangdong, China) (GenBank: MT123290.1) or the B.1.617 (Delta) strains (No. GDPCC, provided by Guangdong Provincial Centre for Disease Control and Prevention, China) in a total of 50 µL volume at day 5 after transduction. All work with SARS-CoV-2 was performed in the Guangzhou Customs District Technology Center’s Biosafety Level 3 (BSL3) laboratories. Two days after the viral challenge, virus titers in lung tissues were measured using the focus-forming assay (FFA), and some lung tissues were fixed for histopathological analysis.

### Focus-forming assay (FFA)

Viral titers were determined using the FFU test. Vero E6 cells (CRL-1586, ATCC) were cultured in RPMI 1640 medium (11875500BT, Gibco) supplemented with 10% fetal bovine serum (FBS), then seeded into a 96-well plate at a density of 1.5 × 10⁴ cells per well overnight and grown into confluent monolayers. The lung tissue was homogenized in 1 mL of sterile PBS with a MagNA Lyser (Roche) before centrifugation at 8,000 rpm for 10 min. The supernatant was collected and used to inoculate the cells for 1 h at 37°C. After removing the inoculum, each well received 100 µL of 1.6% carboxymethylcellulose heated to 37°C. After 24 h, the cells were fixed in 4% paraformaldehyde and permeabilized with 0.2% Triton X-100. The cells were subsequently treated with a rabbit polyclonal antibody against SARS-CoV-2 nucleocapsid protein (NP) (40143-T62, Sino Biological). Next, HRP-labeled goat anti-rabbit secondary antibody (111–035-144, Jackson ImmunoResearch Laboratories) was used. The foci were identified using TrueBlue Peroxidase Substrate (KPL, USA) and counted with an ELISpot reader (Cellular Technology Limited, USA).

### Lung pathology

To investigate lung pathology, lungs from each group were removed and fixed in 4% paraformaldehyde (BL539A, Biosharp) for more than 24 h. The lungs were subsequently embedded in paraffin, sectioned at four microns, and stained with hematoxylin-eosin (H&E) (G1076, Servicebio) following the manufacturer’s instructions. The stained slices were scanned using an upright white light photographic microscope (Eclipse E100, Nikon).

### Statistical analysis

GraphPad Prism software was used for statistical analysis of data and graph generation. Data are expressed as mean ± SEM or SD. Statistical significance was assessed using unpaired two-tailed Student’s test or two-way analysis of variance (ANOVA) with Dunnett’s multiple comparisons test as specified in the corresponding figure legends.

## Data Availability

All source data and data files are available from the authors upon request.

## SUPPLEMENTAL MATERIAL

Supplemental figures jvi.02323-24-s0001.docx Figures S1 and S2.

ASM does not own the copyrights to Supplemental Material that may be linked to, or accessed through, an article. The authors have granted ASM a non-exclusive, world-wide license to publish the Supplemental Material files. Please contact the corresponding author directly for reuse.
