# Supplementary material for: Thymic stromal lymphopoietin improves protective immunity of the SARS-CoV-2 subunit vaccine by inducing dendritic cell-dependent germinal center response
Source: J Virol. 2025 Mar 4;99(4):e02323-24. doi: 10.1128/jvi.02323-24 (PMC11998488; doi:10.1128/jvi.02323-24)
Supplement: Supplemental figures — Figures S1 and S2. [file jvi.02323-24-s0001.docx]

**Supplementary materials**


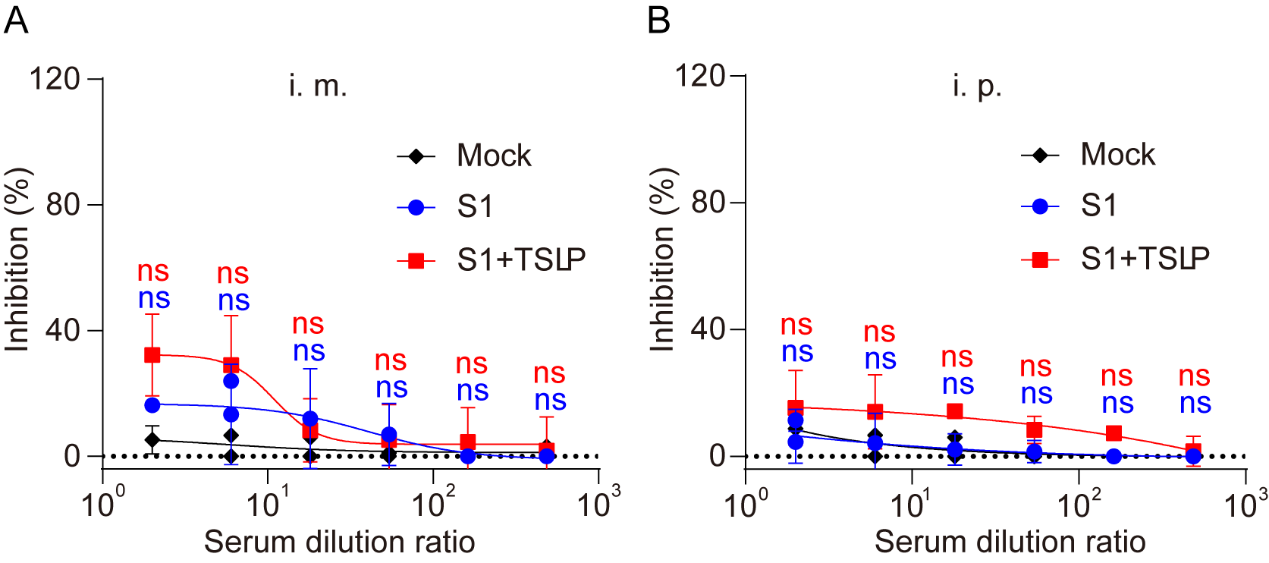


**Fig. S1: TSLP induces poor neutralizing antibodies when the SARS-CoV-2 S1 is administered by intramuscular and intraperitoneal immunization routes.** WT mice were immunized with SARS-CoV-2 S1 (2 μg) in the presence or absence of TSLP (2 μg) via the intramuscular route (i.m.) (A) or intraperitoneal route (i.p.) (B), respectively. Booster immunizations were administered 10 days later. Serum was collected ten days after booster immunization. Unimmunized WT mice were used as the control group (mock). Competitive ELISA was used to determine the percent inhibition of SARC-CoV-2 WT S1 binding to hACE2 in the presence of serially diluted serum (1:2, 1:6, 1:18, 1:54, 1:162, and 1:486 dilution) from mock (black diamond symbol), S1 (blue dot symbol), or S1+TSLP (red square symbol) groups. *n* = 6 mice per group. Data are shown as mean ± SD. ns, no significant difference, by two-way ANOVA with Dunnett's multiple-comparison test. Red asterisks indicate statistically significant differences between S1 and S1+TSLP groups; blue asterisks indicate differences between S1 and mock groups.


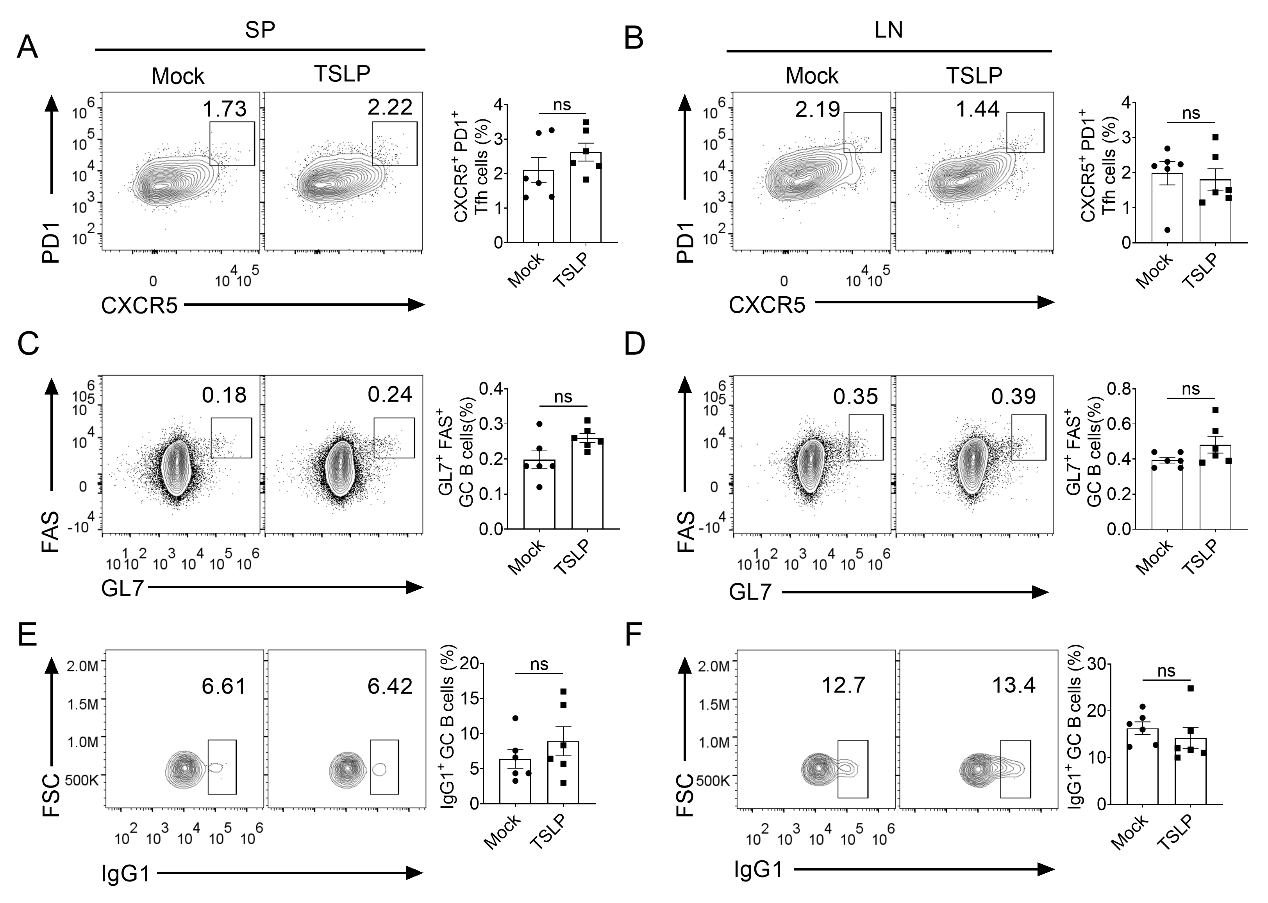


**Fig. S2: TSLP alone does not boost Tfh cells and GC B cell responses after intranasal application.** WT mice (*n* = 6) were received intranasally with TSLP (2 μg) alone. Booster immunizations were performed on 10 and 20 days. Ten days after the second booster immunization, the percentages of CXCR5^+^ PD-1^+^ Tfh cells among live CD19^-^ CD4^+^ CD44^+^ cells (A-B), Fas^+^ GL7^+^ GC B cells among live CD4^-^ CD19^+^ cells (C-D), and IgG1^+^ GC B cells among live CD4^-^ CD19^+^ Fas^+^ GL7^+^ cells (E-F) in the spleen (SP) and draining lymph nodes (LN) were analyzed by flow cytometry. Data are shown as mean ± SEM, ns, no significant difference, by unpaired two-tailed Student’s t-test.
